# Women’s perceptions of contraceptive efficacy and safety

**DOI:** 10.1186/s40834-017-0046-5

**Published:** 2017-06-20

**Authors:** Roshni Kakaiya, Lia L. Lopez, Anita L. Nelson

**Affiliations:** 1 Touro University California, 1310 Club Drive, Mare Island, Vallejo, CA 94592 USA; 2 University of California, Davis, 1 Shields Avenue, Davis, CA 95616 USA; 3 LA BioMed at Harbor UCLA, 1124 W Carson St, Torrance, CA 90502 USA; 4 Western University Health Sciences, 309 E. 2nd Street, Pomona, CA 91766 USA

**Keywords:** Contraceptive attitudes, Contraceptive efficacy, Perception of oral contraceptive health hazards

## Abstract

**Background:**

Adoption of contraceptive implants and intrauterine devices has been less than might be expected given their superior efficacy and convenience. The purpose of this study was to assess knowledge and beliefs held by women, which may influence their contraceptive choices and theirongoing utilization of contraceptive methods.

**Methods:**

English speaking, nonpregnant, reproductive-age women, who were not surgically sterilized, were individually interviewed to obtain limited demographic characteristics and to assess their knowledge about the efficacy of various contraceptive methods in typical use and about the relative safety of oral contraceptives.

**Results:**

A convenience sample of 500 women aged 18–45 years, with education levels that ranged from middle school to postdoctoral level was interviewed. The efficacy in typical use of both combined oral contraceptives and male condoms was correctly estimated by 2.2%; over two-thirds of women significantly *over*estimated the efficacy of each of those methods in typical use. Oral contraceptives were thought to be at least as hazardous to a woman’s health as pregnancy by 56% of women.

**Conclusions:**

The majority of reproductive aged women surveyed substantially overestimated the efficacy of the two most popular contraceptive methods, often saying that they were 99% effective. Women with higher education levels were most likely to overestimate efficacy of oral contraceptives. Women of all ages and education levels significantly overestimated the health hazards of oral contraceptives compared to pregnancy. Overestimation of effectiveness of these methods of contraception, may contribute to lower adoption of implants and intrauterine devices. When individualizing patient counselling, misperceptions must be identified and addressed with women of all educational backgrounds.

**Trial registration:**

Not applicable.

## Background

Contraceptive implants and intrauterine devices (IUDs) provide pregnancy protection on par with permanent contraception. Increased use of these methods has been associated temporally with decreases in unintended pregnancy rates in the United States (US) [1, 2]. Since there appears to be a link between inconsistent contraceptive use and perception of low personal risk for pregnancy, it is interesting to consider that a perception of low pregnancy risk might influence choice of contraceptive method [3]. While combined hormonal contraceptives and progestin-only pills, contraceptive patches, vaginal contraceptive rings and injections offer significant noncontraceptive benefits that are important to many women, inconsistent use of those methods results in first year typical failure rates that are at least 20 times higher than failure rates of IUDs and implants [4]. Male condoms in typical use have been reported to have even higher first year failure rates of 12% [5]. The superior efficacy and convenience of implants and IUDs should appeal to couples, but these top tier methods were still utilized by only 7.3% of US women in the 2011–13 National Survey of Family Growth (NSFG) [6].

Previous surveys and focus groups have tried to determine what various groups (contracepting women, at-risk women and the general public) believe to be the efficacy and safety of various methods of contraception [7–9]. Investigators have also attempted to understand why men and women discontinue contraceptive methods or use them inconsistently [9–16].

There are many factors that enter into a woman’s decision to choose a specific method of birth control, including health restrictions, cost, availability and partner preference. However, surveys consistently report that the most important feature that women want from their contraceptive method is efficacy [17–19]. Given that high priority, we sought to determine if women were aware of the superior pregnancy protection offered by IUDs and implants or if they thought older methods were as effective. We also asked how women rated the health risks of pregnancy compared to the health risks associated with combined oral contraceptives. Finally, we sought to determine if age, education or parity influenced any of the findings.

## Methods

This project was approved by both the John F. Wolf Human Subjects Committee and the Research Committee at the Los Angeles Biomedical Research Institute at Harbor-UCLA Medical Center (LA BioMed) (Project number 30329-01). Requirement for informed consent was waived because the study presented no more than minimal risk to the subjects and no personal identifying information would to be collected.

The study survey first obtained limited demographic information and then asked specific questions about knowledge and beliefs about method efficacy. The survey instrument was beta-tested to determine if the questions were understandable to the population to be studied and to ensure consistency in the way that the two surveyors (RK and LLL) administered the survey. Women were interviewed within the family planning clinic and the gynecology clinic at Harbor-UCLA Medical Center and in the open areas of Los Angeles BioMedical Research Institute campus. In both the gynecology and the family planning clinics, all uninsured women living in the State of California with family incomes below 200% of the Federal poverty levels qualify for enrollment into the state Medicaid program, (Family PACT) which provides all forms of contraception without any charge. Women interviewed at other locations on campus may have had private insurance coverage; at the time of this survey most California insurance companies were providing without charging any co-payments under the provision of the Affordable Care Act. Exclusion criteria for study participation included all of the following: age under 18 or over 45 years, use of permanent contraception (tubal ligation, tubal occlusion, or hysterectomy) pregnancy, and an inability to speak English. Responses gathered about efficacy from these subjects were compared to the estimates derived from the National Survey of Family Growth and published in most standard texts of the time [20]. Specifically, correct estimates of first year failure rates in typical use were assumed to be 9% for oral contraceptives and 18% for male condoms. For purposes of this survey, the term “efficacy” was used because it was more familiar to the women. It is the term used in product labeling and in advertisements for methods. “Efficacy” was calculated as the difference between 100 and the method’s first year failure rate in typical use. Women were offered efficacy choices of 99, 95, 91, 83 and 70% for each of the methods (oral contraceptives, male condoms, IUD, implant). At the conclusion of the survey, women were asked which they thought was more hazardous to a woman’s health—oral contraceptives or pregnancy. The results were compiled into an Excel document; standard statistical programs were used for calculations. Specifically, *p* values were calculated using Graph Pad Quick Calcs with Fisher’s exact test and two tail tests. Chi square tests were applied to percentages of women overall and in each subgroup with answers that were correct vs. incorrect, or correct vs. overestimates or correct vs underestimates. The threshold for statistical significant analyses was set at *p* < 0.05.

## Results

In total, 781 women were individually invited by one of the female researchers (RK, LL) to participate in the survey. Of the 781 women approached, 215 women declined, and 66 women met exclusionary criteria, leaving a study population of 500 women. Virtually all the women who declined to be interviewed expressed an interest in participating, but said that they did not have time available to do so.

The demographic characteristics of the reproductive-aged women in this convenience sample are displayed in Table 1. Their mean age was 25.1 years, ranging from 18 to 45 years slightly over one fifth (20.2%) had only attended high school. One woman’s highest education was middle school. However, over half of the participants had graduated from a 4-year college and another 17% had done graduate work. Parity ranged from 0 to 6; 51.6% of the study population was nulliparous. Given the public setting in which questions were being asked, women were not directly asked about their current sexual activity or contraceptive method use.

**Table 1:** Demographics of Study Population

	Number	Percent (%)
Total	500	100
Age group, years		
Age ≤ 30	270	54
Age >30	230	46
Highest education		
Not high school graduate	24	4.8
High school graduate	77	15.4
Some college	146	29.2
4-year college graduate	168	33.6
Graduate degree	85	17
Parity		
Without children	258	51.6
With children	242	48.4

Table 2 displays the study participants’ estimates of efficacy in typical use of COCs and male condoms. When offered five different estimates of first year efficacy (99, 95, 91, 83, and 70%), 2.2% of the study population correctly identified the first-year efficacies in typical use of both COCs and male condoms. Regardless of age, education or parity, study subjects overwhelmingly overestimated the pregnancy protection provided in typical use of each of these methods. Compared to women with no college experience, those with at least a 4-year college degree were more likely to overestimate the efficacy of COCs (71.1% vs. 55.4% *p* = 0.0028), but not the efficacy of condoms (74.3% vs. 68% *p* = 0.1385). While far fewer women *under*estimated the efficacy of COCs, the less educated women were more likely than women with more education to do so; high school graduates were more likely to underestimate the efficacy of COCs compared to those with college degrees (29.7% vs. 15.2% *p* = .0071). Educational attainment did not affect the proportion of women who underestimated the efficacy of condoms.

**Table 2:** Percent of Women Surveyed Who Estimated Typical Use Efficacy By Category of Response

	Percent of Women						
	Correctly Estimate COC Efficacy	Overestimated Efficacy of COCs	Underestimated Efficacy of COCs	Condoms Correctly Answered	Overestimated Efficacy	Underestimated Efficacy of COCs	Correctly Answered Both Birth Control Pill and Condoms Efficacy
Total Population (*n* = 500)	14.4%	65.0%	19.6%	12.0%	73.0%	16.6%	2.2%
Age (years)							
≤ 30 (*n* = 270)	15.9	63.0	20.0	11.9	71.9	16.3	1.9
> 30 (*n* = 230)	12.6	67.4	19.1	12.2	70.4	17.0	2.6
Education							
≤ High School graduate (*n* = 101)	11.9	55.4	29.7	12.9	67.3	19.8	.99
Some college (*n* = 146)	18.5	60.3	20.5	13.7	68.5	17.1	3.4
≥ 4-year college graduate (*n* = 253)	13.0	71.1	15.2	10.7	74.3	15.0	2.0
Parity							
0 (*n* = 258)	14.0	68.6	16.7	9.5	72.1	14.3	2.3
> 0 (*n* = 242)	14.9	61.2	22.7	13.0	70.2	19.0	2.1

Table 3 shows that 56.2% of all women surveyed believed that oral contraceptives were at least as hazardous to a woman’s health as pregnancy. Almost half (47.7%) of women with at least a 4-year college education said that birth control pills pose at least as great of a risk to a woman’s health as pregnancy, compared to 68.4% of women who had no more than a high school education (*p* = .0001). Parity made no difference; 60% of multiparous women believed that combined hormonal contraceptive pills are at least as risky as pregnancy compared to 52.7% of nulliparous women (*p* = 0.1158).

**Table 3:** Health Risks of COCs vs Pregnancy

	% of Women Responding^a^	
	Pregnancy is Greater Risk	COCs Are at Least as Great a Risk
Total Population (*n* = 473)^a^	43.8	56.2
Age		
≤ 30 (*n* = 261)	44.6	55.4
> 30 (*n* = 222)	42.8	57.2
Education		
≤ HS graduate (*n* = 98)	32.7	67.3
Some college (*n* = 138)	36.2	63.8
≥ 4-year college graduate (*n* = 252)	52.7	47.3
Parity		
0 (*n* = 245)	46.9	53.1
> 0 (*n* = 238)	39.7	60.3

^a^Excludes 37 women who did not answer or were unsure of their response

## Discussion

A woman’s perception of the efficacy and safety of a contraceptive method may strongly influence both her selection of the method and her decision to continue to use it over time [3, 17]. These perceptions are often derived from, or at least perpetuated by, the media and distributed via peer networks, educational facilities, and popular culture, including magazines, television shows, movies, online blogs and other social media [21]. New tools piloted to assess women’s contraceptive knowledge have concluded that contraceptive knowledge may be lower than previous studies have suggested [22].

Despite the importance that women claim they attach to the efficacy of different methods in selecting methods to use, our study found that the vast majority of women was misinformed about the pregnancy protection offered by the most popular methods. Only 2.2% of our study population was able to correctly identify the first-year failure rates in typical use of COCs and the male condoms and most erred on the side of overestimating the efficacy of those methods. These findings may help explain why there has not been a more enthusiastic and widespread adoption of contraceptive implants and intrauterine devices, despite their proven superiority in pregnancy protection [4]. The National Campaign to Prevent Teen and Unintended Pregnancy also showed that most adolescent women believe all contraception is equally effective so that emphasizing the effectiveness of IUDs and implants is not likely to impress young women. They conclude that it is more important to discuss the other attributes and benefits of IUDs and implants than efficacy [23]. A recent national telephone survey of 2302 women veterans, aged 18–44, found that only 50% knew that the male condom was less effective at preventing pregnancy compared to sterilization, IUDs and implants and hormonal contraception [24].

The percent of women who *under*estimated COC efficacy in our study is comparable to findings of the ACOG surveys done in the 1985 and 1993, which found that nearly 4 in 10 women of childbearing age thought that the failure rate of the pill was at least 10% [25]. Surveys of 900 women in Jamaica and India found that only 46% of women knew that COCs are more effective than condoms and only 50% knew that IUDs are more effective than condoms [19]. It is possible that this misinformation contributes to understanding why so many women use methods that are inconsistent with their stated reproductive life plans [26, 27].

Contraceptive users themselves are not alone in these misbeliefs. In a study of parents of 13–17 year olds, Eisenberg et al reported that many parents (especially conservatives) underestimated the perfect use effectiveness of condoms for reducing both pregnancy and STD risks [28]. Parisi et al found that 81% of primary care physicians surveyed underestimated the risk of pregnancy with unprotected intercourse; but 85% overestimated the efficacy of COCs, and 62% overestimated the efficacy of male condoms for pregnancy protection [29].

In the combined 2002 and 2006–2010 National Surveys of Family Growth, 16.5% of sexually active women reported they did not use contraception [30]. Thomas et al have demonstrated that the single most effective step to reduce unintended pregnancy rates in the US would be to increase the proportion of sexually active women not seeking pregnancy who use any method at all [31]. However, it may be easier to persuade women who have decided to contracept to adopt methods that may be more effective. Both approaches are important. Both may benefit from educating women about their risks of pregnancy if they continue their current actions. Since great disparities continue to exist among different racial and ethnic groups independent of access to healthcare, different counselling approaches may be needed [32].

In the televised Nelson congressional hearings in 1970 about oral contraceptive safety, millions of Americans tuned in to hear experts testify that “estrogen is to cancer as fertilizer is to wheat” [33]. Concerns about the safety of contraception are prevalent and long standing. ACOG/Gallop polls conducted in 1985 and 1993 reported that over half of pill users believed they were accepting a significant health risk by taking an oral contraceptive, and over 60% thought that COCs were riskier than childbirth [25]. Recent press coverage of breast cancer and depression risk for a variety of hormonal methods has fueled this underlying mistrust as have ongoing disputes about the clinical significance of potential differences seen in risk of thrombosis [34–38]. National surveys in Ireland in 2010 found that 37% of Irish women agreed with the statement “the OCP has dangerous side effects” and that such agreement was the strongest predictor variable of non-use of OCPs [39]. Excessive fears about the safety of oral contraceptive were also revealed in an earlier survey done by our group and others [39–41].

The clear consensus from all experts is that all of the methods are safer to the health of the women who are candidates than pregnancy [42–45]. This study demonstrates that this last reassuring message has not reached most women. Over half of women interviewed in this study believed that oral contraceptives were at least as hazardous to a woman’s health as pregnancy.

There are important limitations to the generalizability of our results. Very recent calculations of typical use failure rates all lower than the estimates we used in this study. Pill failure rates, in typical use, are now quoted as 6% (down from 9%) and for the male condom, 13% is now quoted [5]. Although the corresponding efficacy rates for those exact estimates (94 and 91%) were not offered as options in this study, it is clear that the findings of overestimation of efficacy still are correct, since so many women estimated 99 and 95% efficacy rates for all methods. The study population represented a diverse sample of English speaking women and included uninsured, indigent women as well as a highly educated medical and research professional staff. However, the study population was geographically isolated. No information was collected about race or ethnicity, so we are not able to assess the impacts of those variables. Rosenfield et al have demonstrated higher rates of contraceptive misinformation among non-Hispanic black women [24]. Direct questions about the subject’s current sexual activity and contraceptive use were not included in the survey as it was administered in a potentially public setting. This may possibly have limited our ability to interpret the differences seen between contraceptive users and nonusers. However, the significant prevalence of misinformation revealed by this study in all women overshadows any of the differences that may have been observed between groups.

Understanding the prevalence of misperceptions about method failures and contraceptive safety can help clinicians provide targeted counselling to enable each woman to make a more informed contraceptive choice.

## Conclusions

This survey of 500 English-speaking reproductive aged, nonpregnant women found that two thirds of women surveyed markedly over-estimated the efficacy of more traditional methods of contraceptive, such as condoms and oral contraceptives. Women with higher education levels were not immune to misinformation; they understated the failure rates at higher rates than women with high school educations only. At the same time, the majority of women overstated the health risks of pill use, reporting that oral contraceptives are at least as hazardous to a woman’s health as pregnancy. Promoting the unparalleled pregnancy protection offered by implants and intrauterine contraceptives may not resonate with potential users because they do not see any room for improvement in efficacy. On the other hand, the increased safety of progestin-only or nonhormonal methods may interest more of them who are apparently quite concerned about the health risks of estrogen-containing pills. Additionally, results demonstrate that women are ill-informed about the potential health risks of pregnancy.

## Abbreviations

ACOG: American Congress of Obstetricians and Gynecologists
COCs: Combined oral contraceptives
IUD: Intrauterine device
OCP: Oral contraceptive pill
